# Supplementary material for: N-palmitoyl-D-glucosamine, A Natural Monosaccharide-Based Glycolipid, Inhibits TLR4 and Prevents LPS-Induced Inflammation and Neuropathic Pain in Mice
Source: Int J Mol Sci. 2021 Feb 2;22(3):1491. doi: 10.3390/ijms22031491 (PMC7867376; doi:10.3390/ijms22031491)
Supplement: Supplementary file 1 [file ijms-22-01491-s001.pdf]

-Supplementary Materials

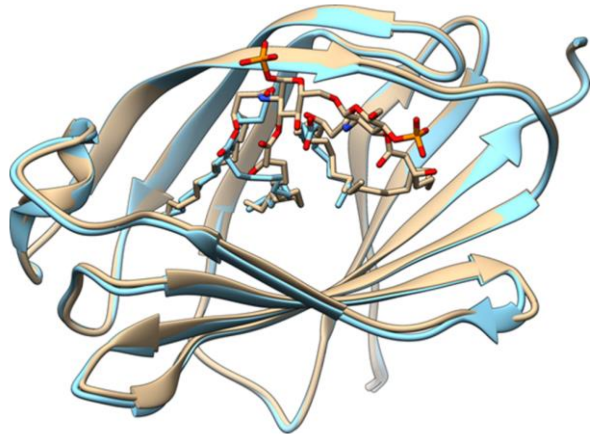

Figure S1. X-ray structures of MD-2 in complex with lipid IVa (PDB id: 2E59), colored in tan, and myristic acid (PDB1 id: 2E56), colored in sky blue, after best fit at protein backbone level. Oxygen and nitrogen atoms are colored in red and blue, respectively. Ligands are shown in stick representation.

A

Gene name	RAW264	HEK293	HELA
TLR4	24.54±0.05	N.D.	29.63±0.02
S16	16.73±0.05	17.72±0.03	16.95±0.07

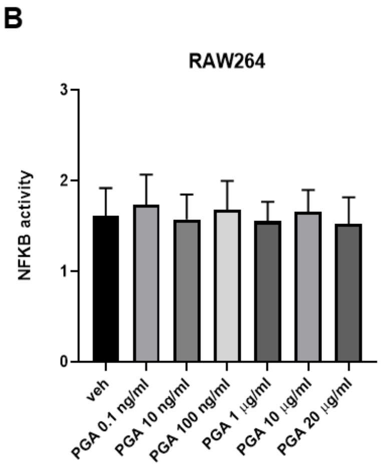

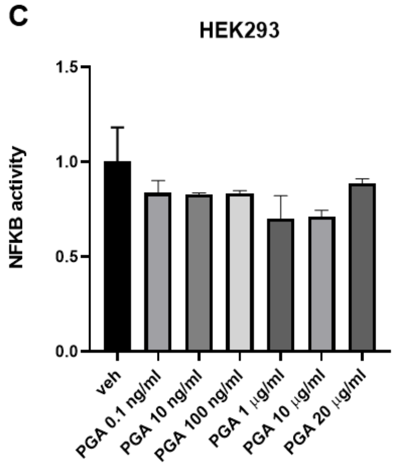

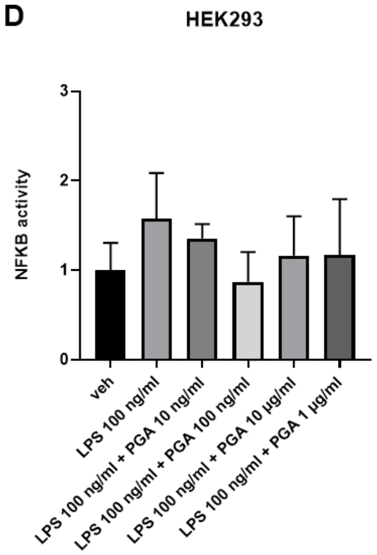

Figure S2. TLR4 expression measurement and luciferase assays performed in non-transfected RAW264 and HEK293 cells. (**A**) The table shows the cycle threshold (Ct) value (mean of 4 independent experiments ± SEM) of TLR4 measured in RAW264 and HEK293 cells. Hela cells served as a control for the human TLR4 primer sequences. N.D. = signal not detected (**B**) Luciferase activity measured in

relative light units (RLU) normalized to the vehicle group was measured in non-transfected RAW264; Luciferase activity measured in relative light units (RLU) normalized to the vehicle group was measured in non-transfected (**C**) and TLR4 transfected **(D)** HEK293 cells stimulated with the indicated concentrations of PGA.

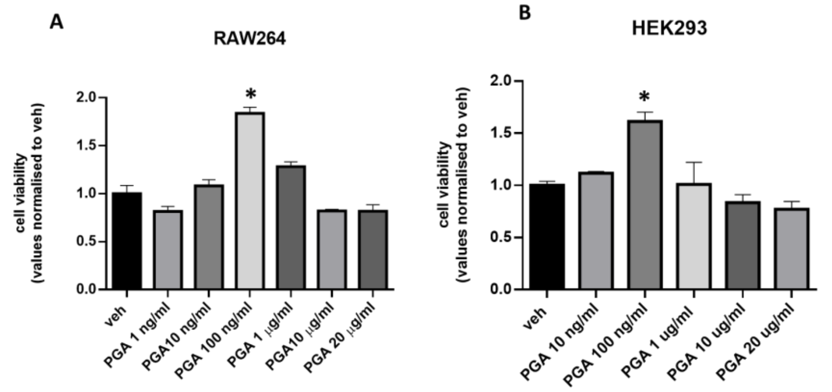

Figure S2. Cell viability was assessed using the MTT assay in RAW264 (**A**) and HEK293 (**B**) cells. Values are mean ± SEM; n = 4; The asterisk denotes a * $p < 0.05$ versus veh group; one-way ANOVA with Tukey's test was used to assess significance.

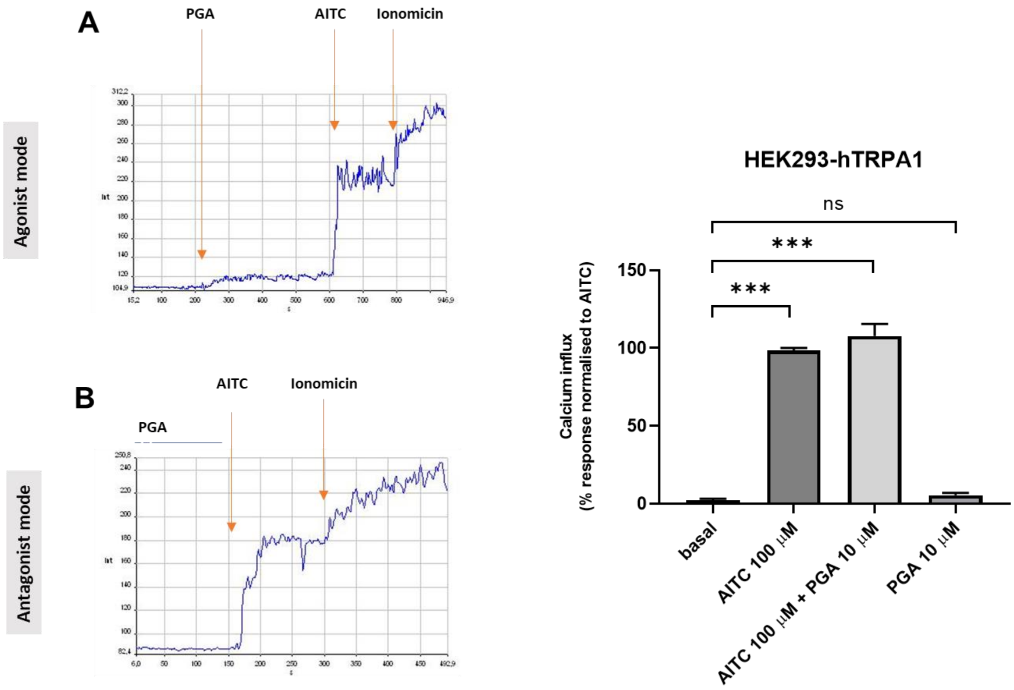

Figure S4. Effect of PGA in TRPA1-transfected HEK293 cells. (**A**,**B**) Representative traces showing the effect of PGA 10 μM, Allyl isothiocyanate (AITC) 100 μM and Ionomicin 4 μM in human embryonic kidney 293 (HEK293) cells stably transfected with rat TRPA1. (**C**) Bar Graph showing quantification of results. Values are mean ± SEM of three independent determinations; *** denotes a $p \leq 0.01$; one-way ANOVA with Tukey's test was used to assess significance. Data are expressed as % relative to AITC 100 μM. ns: not significant.
